# Body Mass Index and Primary Headache: A Hospital-Based Study in China

**DOI:** 10.1155/2019/4630490

**Published:** 2019-04-15

**Authors:** Qingqing Huang, Huiqing Yu, Ningning Zhang, Bingling Guo, Changyan Feng, Shiqiang Wang, Xiping Liang

**Affiliations:** Chongqing University Cancer Hospital & Chongqing Cancer Institute & Chongqing Cancer Hospital, China

## Abstract

**Objective:**

Primary headache and obesity are highly prevalent disorders in the general population. Although many studies have reported an association between the two, there is still no overall comprehension about this relationship. To gain a more accurate understanding in this regard, we analyzed data from a 2011 cross-sectional study in Chongqing, China.

**Methods:**

Patients with a chief complaint of headache were administered a headache questionnaire and diagnosed by neurology doctors in accordance with the International Classification of Headache Disorders 2nd Edition (ICHD-II) criteria. Patients aged < 18 years or diagnosed with secondary headache were excluded.

**Results:**

Of 1327 patients who cited headache as the chief complaint, 16 were excluded for missing data, while 396 were diagnosed with chronic headache (177 chronic migraine [CM], 186 chronic tension-type headache [CTTH], and 33 other chronic headache) and 915 with episodic headache (369 episodic migraine [EM], 319 episodic tension-type headache [ETTH], and 227 other episodic headache). Chronic headache patients had a higher number of headache days per month, longer duration of headache history, and greater tendency to overuse analgesics than episodic headache patients. The CM and ETTH patients were more apt to be overweight and had a significantly greater body mass index (BMI; p < 0.05) than the EM and CTTH patients. Overweight (odds ratio [OR] = 3.64; 95% confidence interval (CI), 1.19–8.81) and obesity (OR = 28.63; 95% CI, 2.96–276.6) were independently associated with CM but not with other headaches, and this association was not influenced by other factors such as medication overuse.

**Conclusions:**

The relationship between headache and overweight/obesity varies depending on the type of primary headache. CM patients are more likely to have a higher body mass index than EM patients, while ETTH patients are more likely to be overweight/obese than CTTH patients.

## 1. Introduction

Headache, one of the most frequent reasons for consultation at neurology clinics, causes disability and impairment of quality of life. More than 50% of adults in European countries indicated that they had suffered from headaches within the past year [1]; in China, the estimated 1-year prevalence of primary headache disorders was reported to be 23.8% [2].

Obesity is often comorbid with a number of chronic headache syndromes. Individuals with episodic headache and obesity are five times more likely to develop chronic daily headache (CDH) than normal-weight individuals [3]. A cross-sectional population study confirmed the relationship between obesity and CDH and suggested that obesity was a risk factor for the transformation of episodic migraine (EM) to chronic migraine (CM) [4]. After adjusting for age, sex, race, and education, Scher and colleagues found that obesity was associated with prevalent CDH (odds ratio [OR] = 1.34) [5]. In addition, episodic headache progressed to CDH far more frequently among obese patients than among normal-weighted patients [5].

In this study, we analyzed primary headache and its subtypes in order to (1) analyze the association of body mass index (BMI) with headache (episodic and chronic); (2) separately compare patients with episodic and chronic migraine/tension-type headache (TTH)/other headache types; and (3) analyze the BMI of migraine and TTH patients after adjusting for confounding factors.

## 2. Methods

The present study involved analysis of data from a previous study [6]. The target population for this study included adults aged 18 years or above who visited the neurology outpatient department at the First Affiliated Hospital of Chongqing Medical University, China, in 2011 with a chief complaint of headache. The demographic characteristics and clinical features of the headache (including frequency, duration, intensity, and other symptoms) were recorded by using a headache questionnaire [6]. At their first visit, all headache patients underwent funduscopy and physical neurological examination in our clinic. When necessary, electroencephalography, radiography, transcranial Doppler ultrasound, and brain computed tomography or magnetic resonance imaging were also performed in order to exclude secondary headache, including idiopathic intracranial hypertension. Patients with abnormal findings in these examinations were excluded from the present study; such patients were asked to stay in the hospital and undergo lumbar puncture and intracranial pressure measurement.

The intensity of the most frequent headache in the last 3 months was assessed by using an 11-point pain scale (0, no pain; 1–3, mild pain; 4–6, moderate pain; and 7–10, severe pain). Body height and weight and waist circumference were measured with the subjects wearing light clothing and no shoes.

BMI was calculated by using the following formula: BMI (kg/m^2^) = weight (kg) / height (m^2^). The patients were grouped into five categories on the basis of BMI in accordance with the World Health Organization (WHO) guidelines: underweight (<18.5 kg/m^2^); normal weight (18.5 to <25 kg/m^2^); overweight (25 to <30 kg/m^2^); obese (30 to 34.9 kg/m^2^); and morbidly obese (≥35 kg/m^2^). Because of the small number of morbidly obese patients, they were grouped with obese patients in a single group.

On the basis of the ICHD-II guidelines, primary headache was classified into three subtypes: migraine, TTH, and other headaches. We categorized other primary headache types, such as neuralgia, needle-like headache, and trigeminal autonomic cephalalgias, as other headaches because the sample size for each of these headaches was too small to analyze.

In accordance with the duration of headache and mean headache days per month over the last 3 months, the patients were assigned to the episodic (EM, episodic TTH [ETTH], or other episodic headache) or chronic (CM, chronic TTH [CTTH], or other chronic headache) headache groups. Patients who experienced headaches for at least 15 days per month for at least 3 months, thus fulfilling the ICHD-II criteria, were diagnosed with chronic headache (CM/CTTH/other chronic headache) [7]. The data of the migraine and TTH groups were submitted to multiple logistic regression analysis in order to adjust for confounding factors.

Medication overuse was diagnosed in accordance with the following criteria: headaches for 15 days or more per month, developed as a consequence of regular overuse of acute or symptomatic headache medications (on 10 or more days or 15 or more days per month, depending on the medication) for more than 3 months.

The study protocol was reviewed and approved by the ethics committee of the First Affiliated Hospital of Chongqing Medical University. The patients were informed of the purpose of the study, and they provided consent before participating in this study.

## 3. Statistical Analysis

Statistical analyses were performed by using SPSS version 23.0. Data were summarized by using frequency counts and descriptive statistics. Measurement variables were expressed as mean ± standard error of the mean. Intergroup comparisons were performed by one-way analysis of variance, t test, or multiple logistic regression analysis, as appropriate. A value of p < 0.05 was considered statistically significant.

## 4. Results

### 4.1. Demographic and Clinical Characteristics of the Episodic and Chronic Headache Patients

During the study period, 10,315 patients visited the neurology outpatient department of our hospital, of whom 1327 patients (12.9%) had cited headache as their chief complaint. Among the 1327 patients, exact information on body height and weight was available for only 1311 patients. Of the 1311 patients, 396 were diagnosed with chronic headache (CM, 177; CTTH, 186; and other chronic headaches, 33) and 915 with episodic headache (EM, 369; ETTH, 319; and other episodic headaches, 227).

In the episodic and chronic headache groups, the proportion of women was greater than that of men, and there was no significant intergroup difference in this regard (68.5% vs. 73.4%; p = 0.075; Table 1). Compared with the episodic headache patients, those with chronic headache were older (43.41 ± 13.05 years vs. 46.92 ± 12.51 years; p < 0.001), less educated (p < 0.001), and more apt to overuse analgesics; they also had a greater number of headache days per month and a longer duration of headache history. With 70.1% of the chronic headache patients having received primary school education or less, the rates of high school/technical school and university education in this group were lower than those in the episodic headache group. There was no significant difference in BMI or the distribution of the four BMI subgroups between the episodic and chronic headache groups (p > 0.05).

### 4.2. Association between BMI and the Three Primary Headache Types

In order to have a better understanding of the relationship between headache and BMI, we further analyzed the three subtypes of primary headache separately (Tables 2 and 3).

Among the migraineurs, the proportion of women was greater than that of men, with no significant difference in this regard between the CM and EM groups (77.5% vs. 82.7%; p = 0.176). Compared with the EM patients, the CM patients had a lower education level (p < 0.001), and a greater proportion of them were married (p = 0.001). The BMI of the CM group was significantly higher (21.90 ± 2.88 vs. 23.34 ± 2.34; p < 0.001) than that of the EM group.

The episodic and chronic TTH patients showed similar trends as the migraineurs in terms of age, sex, education level, and pain intensity (Table 3), although the intergroup differences were not significant (p > 0.05). There were no significant differences between the two groups in marital status, smoking habit, or alcohol consumption. However, ETTH patients had a higher BMI than CTTH patients (22.94 ± 2.95 vs. 22.21 ± 3.24; p = 0.012).

There was no statistically significant difference between the other episodic and chronic headache groups except in pain intensity. There was no significant difference in BMI or distribution of the four BMI subgroups between these two groups (p > 0.05; data not shown).

**Figure 1:**
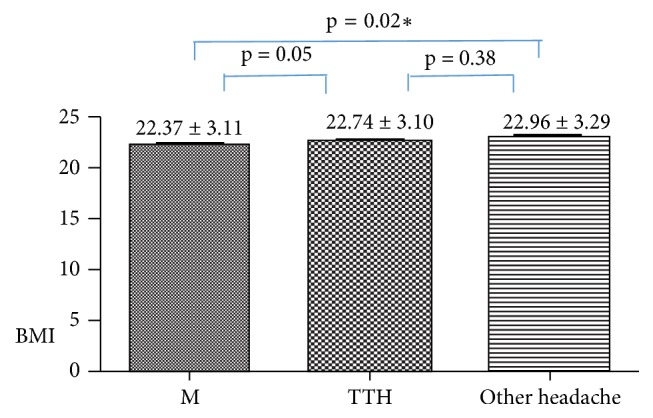
Results of multiple comparisons of BMI among the migraine/TTH/other headache groups. Note. *∗* means that the BMI of the migraineurs was lower than that of the other two groups. However, the difference reached statistical significance only between the migraine and other headache groups. BMI: body mass index; M: migraine; TTH: tension-type headache.

We performed multiple comparisons of BMI among the migraine, TTH, and other headache groups. The migraineurs had lower BMI than TTH and other headache patients; however, the difference was statistically significant only between the migraine and other headache groups (p = 0.018). The difference in BMI between the migraineurs and TTH patients (p = 0.05), or the TTH and other headache patients, (p > 0.05) was not significant (Figure 1).

Finally, we compared the proportions of patients with abnormal and normal BMI in the three primary headache groups (Table 4). Among the migraineurs, the proportions of obese and overweight patients were greater in the CM group than in the EM group, with only the difference in the number of overweight patients being significant (EM vs. CM: 12.4% vs. 31.9%; p < 0.001).

Among the TTH patients, the proportion of overweight patients in the CTTH group was lower than that in the ETTH group (ETTH vs. CTTH: 26.4% vs. 17.5%; p = 0.018). There was no statistically significant difference in this regard between episodic and chronic patients in the other headaches group (p > 0.05; data not shown).

### 4.3. Multiple Logistic Regression Analysis of Migraine and TTH

Among the three primary headache types, only CM and ETTH were associated with BMI, especially in case of overweight patients. However, there were some other factors that might have influenced this association. We, therefore, evaluated the migraine and TTH groups by logistic regression analysis (Tables 5 and 6).

The results demonstrated that BMI was independently associated with CM (p = 0.005) but not with CTTH. Relative to normal weight, the odds ratios for overweight and obesity were 3.24 (95% CI, 1.19–8.81) and 28.63 (95%CI, 2.96–276.6) in CM patients. The relationship between BMI and headache was not influenced by medication overuse. The results of logistic regression analysis showed that the odds ratios for CM and CTTH were 0.01 (95% CI = 0.001–0.035) and 0.044 (95% CI = 0.01–0.203), respectively.

## 5. Discussion 

Both headache and obesity are highly prevalent disorders in the general population, and their relationship has been investigated in clinical studies in recent years. Headache is often accompanied by abnormal BMI, especially obesity. Low BMI (underweight) is associated with the least likelihood of headache, and the risk of headache increases significantly with increase in BMI [8]. Ohayon also reported that overweight/obese respondents are more likely to report morning headache than adults with normal BMI [9]. Additionally, in a sample of 15,000 Australian women, Brown and colleagues also found that obese persons were more likely to report a headache than normal-weight individuals (OR = 1.47) [10].

The relationship between underweight and headache was uncertain. In our study, underweight had no association with primary headache. This is similar to the findings of another study which reported that migraine is not significantly associated with BMI < 18.5 kg/m^2^[11]. Keith et al. also found that BMI < 20 kg/m^2^ (5th percentile, approximately) was associated with the least likelihood of headache among US women in 11 datasets [8]. However, in four datasets, they observed some evidence indicating that unusually low BMI might be associated with an increased risk for headache. This study revealed a J-shaped relationship between BMI categories and headache: increased BMI might be associated with a decreased risk of headache among women with BMI < 20 kg/m^2^ and an increased risk of headache among those with BMI > 20 kg/m^2^.

The main findings of our study were as follows: (1) Compared with episodic headache patients, chronic headache patients were less educated, older, and more likely to overuse analgesics and also had a longer duration of headache history. However, there was no significant difference in BMI or the distribution of the four BMI subgroups between the two groups. (2) CM and ETTH patients had a higher BMI than EM and CTTH patients; however, there was no statistically significant difference in this regard among patients with other episodic and chronic headaches. (3) After adjustment for age, sex, marriage, education level, and other clinical factors, overweight and obesity were associated with CM but not with CTTH.

Migraineurs usually have some gastrointestinal symptoms, such as nausea and vomiting. They also experience aggravation of migraine because of routine physical activity, and migraine attacks often influence their appetite. In adults with migraine, high BMI is associated with more frequent and severe migraine attacks [12], and the prevalence of migraine is higher among underweight and obese participants than among those with normal BMI [13]. Some studies have reported that obesity is related to migraine [3, 14, 15], and the risk of EM progressing to CDH increases with obesity [5, 16] only in case of migraineurs and not in CTTH patients [4, 17]. These previous findings are consistent with those of our study.

However, a few studies have reported contradictory results, having found no significant relationship between migraine and abnormal BMI [18–20]. In a previous study, migraine was not significantly associated with BMI < 18.5 or 30 kg/m^2^ [11]. In other studies, BMI was not associated with the prevalence of migraine [12, 21]. A Swedish study involving 684 women between the ages of 45 and 74 years found no significant association between obesity and the prevalence of migraine or the frequency, intensity, or duration of migraine attacks [22].

In our study, CM patients were more likely to be obese/overweight, while the proportion of overweight patients in the CTTH group was lower than that in the ETTH group. Recent findings based on data from 30,703 episodic headache cases revealed a similar trend: obesity was associated with an increased frequency of headaches and increased disability among patients with migraines but not among patients with severe tension-type headaches [14].

Some preventive drugs, such as topiramate, flunarizine, and amitriptyline, might be associated with weight gain or loss [23, 24], thus influencing the BMI of headache patients. However, the proportion of preventive drugs used in the present study was low. Our previous data [6] showed that symptomatic/acute drugs such as acetaminophen and aspirin—but not preventive drugs—were the most commonly used drugs. Prophylaxis medications were usually taken by patients upon advice of a doctor after their first interview in the clinic. Therefore, we did not take preventive drugs into account for analysis in the present study.

In our study, overweight, and not obese, was the most common classification among patients with abnormal BMI; we speculate that this is because of constitutional differences. Asians are less likely to be obese or morbidly obese than Europeans in accordance with the WHO criteria. The criteria of weight for adults proposed by the Chinese National Health and Family Planning Commission [25] define obesity as BMI ≥ 28, but they do not define morbid obesity; the BMI values defined in these criteria are lower than those described in the WHO criteria. Therefore, the proportion of obese or morbidly obese patients in our study was lower than that described in the WHO criteria.

Some clinical studies have evaluated the association between weight loss and headache frequency. In a small prospective observational study by Bond et al.[26], migraine frequency, pain severity, and disability were significantly reduced after substantial weight loss through bariatric surgery. Moreover, patients who experienced greater weight loss were more likely to experience a 50% or greater decrease in headache frequency [26]. Similar results were observed by Novack et al. [27] in 29 obese women with migraine following bariatric surgery. A study by Verrotti et al. reported that decrease in BMI is associated with a decrease in migraine and migraine outcomes [15].

Few studies have evaluated the association between BMI and TTH. In our study, CM patients were more likely to have a higher BMI than EM patients, while ETTH patients were more likely to be overweight/obese than CTTH patients. A previous population study also found that obesity is associated with CM but not with CTTH [4]. Another study found that BMI is not associated with headache frequency or disability of headaches among individuals with severe ETTH [28].

The mechanisms responsible for the association of obesity with migraine and TTH are unclear. Some scholars believe that sympathetic dysfunction plays an important role in this association. Migraine is a disorder of sympathetic dysfunction [29, 30], being characterized by an increase in the release of sympathetic cotransmitters such as dopamine, prostaglandins, adenosine triphosphate, and adenosine. In a previous study, the plasma levels of dopamine and norepinephrine in CM patients were found to be severalfold higher than those in healthy control subjects (p > 0.001) [31]. Patients with migraine show hyperresponsive sympathetic vasomotor activity during standing [32]. Sympathetic tone, which is evaluated on the basis of resting heart rate, is higher in migraine and CTTH patients than in ETTH patients [33]. This might be one of the reasons why the migraineurs had lower BMI than the other primary headache patients in the present study and also why EM and CTTH patients had a lower BMI than CM and ETTH patients.

Beta-blockers, which selectively bind to *β*-adrenergic receptors in the sympathetic nervous system and thus antagonize the excitatory effect of neurotransmitters and catecholamines, are usually used for preventive treatment of migraine.

Additionally, chronic inflammation, adipokines, sex hormones, and psychiatric factors might play a role in the development of chronic headache. Further studies are required to gain a more comprehensive understanding of the mechanism underlying the association between BMI and chronification of headache, which might aid headache prevention, treatment, and investigation.

Finally, our study has some limitations. First, this was a clinic-based study, and all participants were patients who visited our hospital for treatment. Second, this was a cross-sectional study with a limited study sample. Therefore, a follow-up study is needed to confirm our findings. Third, this study was conducted at a single center, which might be a potential source of bias. Therefore, the results might not be applicable to the general population. Further studies should be conducted among the general population in the future.

## 6. Conclusion

Migraine patients had lower BMI scores than those with other primary headaches. However, compared with EM and CTTH patients, those with CM and ETTH were more likely to be overweight and obese. Obesity/overweight plays an important role in the chronification of migraine. It is possible that sympathetic dysfunction and other mechanisms play an important role in the association between headache and obesity/overweight, and our findings support this theory. Further investigations are needed to better understand this mechanism.

Our findings demonstrating that overweight/obesity is independently associated with CM but not with CTTH are significant for clinicians. Further research is needed in the future to confirm these relationships.

## Figures and Tables

**Table 1:** Demographic and clinical characteristics of the episodic and chronic headache groups.

Variable	Episodic headache (n = 915)	Chronic headache (n = 396)	p value
Sex			
Female (n, %)	632 (68.5%)	287 (73.4%)	0.075
Female to male ratio	2.18	2.76	
Age (years, mean ± SD)	43.41 ± 13.05	46.92 ± 12.51	<0.001
Marriage (n, %)			0.301
Married	798 (86.7%)	353 (90.3%)	
Unmarried	98 (10.7%)	30 (7.7%)	
Divorced	6 (0.7%)	3 (0.8%)	
Widowed	18 (2%)	5 (1.3%)	
Education level (n, %)			<0.001
Primary school or less	501 (55.4%)	269 (70.1%)	
High school or technical school	197 (21.8%)	67 (17.4%)	
University	207 (22.9%)	48 (12.5%)	
Elevated blood pressure (n, %)	203 (22.6%)	96 (25.4%)	0.287
Smoking (n, %)	141 (16.9%)	67 (18.9%)	0.414
Alcohol consumption (n, %)	43 (5.3%)	25 (7.1%)	0.207
Medication overuse	8 (0.9%)	133 (34%)	<0.001
Family headache history (n, %)	333 (38%)	151 (40.5%)	0.412
Duration of headache history (years, mean ± SD)	6.85 ± 9.41	11.23 ± 11.31	<0.001
Pain intensity (mean ± SD)	4.41 ± 2.22	4.14 ± 2.30	0.048
Average headache days/month (days, mean ± SD)	3.08 ± 5.02	22.73 ± 9.14	<0.001
BMI (mean ± SD)	22.57 ± 3.07	22.76 ± 3.02	0.311
BMI classification			0.163
Underweight	68 (7.4%)	28 (7.2%)	
Normal weight	669 (72.7%)	266 (68%)	
Overweight	169 (18.4%)	86 (22%)	
Obese*∗*	14 (1.5%)	11 (2.8%)	
Waist/height ratio	0.45 ± 0.12	0.45 ± 0.12	0.951

Note: ^*∗*^There were only two morbidly obese patients; they were, therefore, grouped together with the obese patients. SD: standard deviation.

**Table 2:** Comparison of BMI and other variables in the migraine group: EM vs. CM.

Variable	EM (n = 369)	CM (n =177)	p value
Female (n, %)	289 (77.5%)	143 (82.7%)	0.176
Age (years, mean ± SD)	40.71 ± 20.34	46.9 ± 16.89	<0.001
Marriage (n, %)			0.001
Married	304 (82.4%)	164 (92.7%)	
Single^#^	65 (17.6%)	13 (7.3%)	
Education level (n, %)			<0.001
Primary school or less	192 (52.7%)	127 (75.6%)	
High school or technical school	81 (22.3%)	28 (16.7%)	
University	91 (25%)	13 (7.7%)	
Elevated blood pressure (n, %)	68 (18.9%)	50 (29.8%)	0.005
Smoking (n, %)	40 (11.4%)	25 (15.3%)	0.211
Alcohol consumption (n, %)	17 (4.9%)	15 (9.2%)	0.06
Pain intensity (mean ± SD)	4.5 ± 2.40	4.4 ± 2.62	0.714
Medication overuse	2 (2.1%)	94 (97.9%)	<0.001
Average headache days/month (days, mean ± SD)	3.94 ± 5.18	21.79 ± 8.95	<0.001
Family headache history (n, %)	183 (68.8%)	83 (31.2%)	0.385
Duration of headache history (years, mean ± SD)	10.83 ± 10.60	14.74± 11.98	<0.001
BMI (mean ± SD)	21.90 ± 2.88	23.34 ± 2.34	<0.001
Waist/height ratio	0.45 ± 0.08	0.45 ± 0.11	0.682

Notes: ^#^Single patients included unmarried, divorced, and widowed individuals. SD: standard deviation; EM: episodic migraine; CM: chronic migraine.

**Table 3:** Comparison of BMI and other variables in the TTH group: ETTH vs. CTTH.

Variable	ETTH (n = 319)	CTTH (n = 186)	p value
Female (n, %)	197(61.8%)	123 (66.1%)	0.325
Age (years, mean ± SD)	45.49 ± 12.52	46.84 ± 13.76	0.272
Marriage (n, %)			0.357
Married	291 (91.2%))	165 (88.7%)	
Single^*∗*^	28 (8.8%)	21 (11.3%)	
Education level (n, %)			0.533
Primary school or less	189 (60%)	119 (64.3%)	
High school or technical school	65 (20.6%)	37 (20%)	
University	61 (19.4%)	29 (15.7%)	
Elevated blood pressure (n, %)	86 (27.2%)	39 (21.5%)	0.161
Smoking (n, %)	63 (21.7%)	36 (21.8%)	0.981
Alcohol consumption (n, %)	16 (5.7%)	8 (4.9%)	0.73
pain intensity (mean ± SD)	3.97 ± 1.88	3.87 ± 2.03	0.584
Medication overuse (n, %)	4 (1.3%)	37 (19.9%)	<0.001
Average headache days per month (days, mean ± SD)	1.67 ± 9.03	11.81 ± 28.69	<0.001
Family headache history (n, %)	96 (31.4%)	62 (34.1%)	0.3
Duration of headache history (years, mean ± SD)	4.60 ± 7.07	9.05 ± 10.27	<0.001
BMI (mean ± SD)	22.94 ± 2.95	22.21 ± 3.24	0.012
Waist/height ratio	0.44 ± 0.13	0.44 ± 0.13	0.975

Notes. ^*∗*^Single patients included unmarried, divorced, and widowed individuals. SD: standard deviation; ETTH: episodic tension-type headache; CTTH: chronic tension-type headache.

**Table:** (a) Migraine

Variable	EM (n = 369)	CM (n =177)	p value
Compared with normal BMI			
Underweight	35 (10.8%)	9 (7.5%)	0.198
Overweight	41 (12.4%)	52 (31.9%)	<0.001
Obese	4 (1.4%)	5 (4.3%)	0.078

**Table:** (b) TTH

Variable	ETTH (n = 319)	CTTH (n = 186)	p value
Compared with normal BMI			
Underweight	20 (8.1%)	16 (10.2%)	0.296
Overweight	81 (26.4%)	30 (17.5%)	0.018
Obese	4 (1.7%)	5 (3.4%)	0.24

EM: episodic headache; CM: chronic headache; ETTH: episodic tension-type headache; CTTH: chronic tension-type headache.

**Table 5:** Multiple logistic regression analysis of the CM group.

Variable	p value	OR	95% CI for OR
Age	0.46	1.02	0.98–1.05
Marriage	0.90	0.92	0.22–3.74
Education level	0.71	0.89	0.5–1.61
Elevated blood pressure	0.76	0.86	0.31–2.37
Medication overuse	<0.001	0.01	0.001–0.035
Average headache days per month	<0.001	1.27	1.21–1.33
Duration of headache history	0.54	0.99	0.95–1.03
BMI classification	0.005		
Compared with normal weight			
Underweight	0.699	0.71	0.12–4.14
Overweight	0.021	3.24	1.19–8.81
Obese	0.004	28.63	2.96–276.6

CM: chronic migraine; OR: odds ratio; CI: confidence interval; BMI: body mass index.

**Table 6:** Multiple logistic regression analysis of the TTH group.

Variable	p value	OR	95% CI for OR
Medication overuse	<0.001	0.044	0.01–0.203
Average headache days per month	<0.001	1.233	1.19–1.28
Duration of headache history	0.053	1.036	1.0–1.07
BMI classification	0.361		
Compared with normal weight)			
Underweight	0.403	1.679	0.5–5.66
Overweight	0.154	0.54	0.23–1.26
Obese	0.736	1.46	0.16–13.45

TTH: tension-type headache; OR: odds ratio; CI: confidence interval; BMI: body mass index.

## Data Availability

The data used to support the findings of this study are available from the corresponding author upon request.

## Abbreviations

BMI: Body mass index
CM: Chronic migraine
EM: Episodic migraine
ETTH: Episodic tension-type headache
TTH: Tension-type headache
CTTH: Chronic tension-type headache
ICHD: International Classification of Headache Disorders
CDH: Chronic daily headache
OR: Odds ratio.
